# Pediatric high-impact conditions in the United States: retrospective analysis of hospitalizations and associated resource use

**DOI:** 10.1186/1471-2431-12-61

**Published:** 2012-06-08

**Authors:** Rebecca L Miller, Achamyeleh Gebremariam, Folafoluwa O Odetola

**Affiliations:** 1 Department of Pediatrics and Communicable Diseases, University of Michigan Health System, Ann Arbor, Michigan, 48109, USA; 2 Department of Pediatrics and Communicable Diseases, Division of Pediatric Critical Care Medicine, University of Michigan Health System, Ann Arbor, Michigan, 48109, USA; 3 Department of Pediatrics and Communicable Diseases, Child Health Evaluation and Research Unit, University of Michigan Health System, Ann Arbor, Michigan, 48109, USA; 4 6C07, 300 North Ingalls Street, Ann Arbor, Michigan, 48109, USA

## Abstract

**Background:**

Child mortality in the United States has decreased over time, with advance in biomedicine. Little is known about patterns of current pediatric health care delivery for children with the leading causes of child death (high-impact conditions). We described patient and hospital characteristics, and hospital resource use, among children hospitalized with high-impact conditions, according to illness severity.

**Methods:**

We conducted a retrospective study of children 0–18 years of age, hospitalized with discharge diagnoses of the ten leading causes of child death, excluding diagnoses not amenable to hospital care, using the 2006 version of the Kid’s Inpatient Database. National estimates of average and cumulative hospital length of stay and total charges were compared between types of hospitals according to patient illness severity, which was measured using all-patient refined diagnosis related group severity classification into minor-moderate, major, and extreme severity.

**Results:**

There were an estimated 3,084,548 child hospitalizations nationally for high-impact conditions in 2006, distributed evenly among hospital types. Most (84.4%) had minor-moderate illness severity, 12.2% major severity, and 3.4% were extremely ill. Most (64%) of the extremely ill were hospitalized at children’s hospitals. Mean hospital stay was longest among the extremely ill (32.8 days), compared with major (9.8 days, p < 0.0001), or minor-moderate (3.4 days, p < 0.001) illness severity. Mean total hospital charges for the extremely ill were also significantly higher than for hospitalizations with major or minor-moderate severity. Among the extremely ill, more frequent hospitalization at children’s hospitals resulted in higher annual cumulative charges among children’s hospitals ($ 7.4 billion), compared with non-children teaching hospitals ($ 3.2 billion, p = 0.023), and non-children’s non-teaching hospitals ($ 1.5 billion, p < 0.001). Cumulative annual length of hospital stay followed the same pattern, according to hospital type.

**Conclusion:**

Gradation of increasing illness severity among children hospitalized for high-impact conditions was associated with concomitantly increased resource consumption. These findings have significant implications for children’s hospitals which appear to accrue the highest resource use burden due to preferential hospitalization of the most severely ill at these hospitals.

## Background

Child mortality has decreased over the last several decades as scientific advancement has allowed the treatment of previously lethal disease. Hospitals now care for a population of children that requires more invasive procedures, innovative technology and multidisciplinary team approach to delivery of care. In an era of scarce health care resources and need for increased accountability, it is important to characterize pediatric health care resource use as it varies by patient and hospital characteristics, to develop health care delivery models that optimize efficiency of care and patient outcomes.

Little is known about patterns of current pediatric health care delivery for children with the leading causes of child death in the United States [1]. These conditions, herein referred to as *high-impact conditions* because of their significant public health impact, include: perinatal conditions, congenital anomalies, chronic lower respiratory disease, influenza & pneumonia, trauma, malignant neoplasms, heart disease, benign neoplasms, sepsis, and cerebrovascular disease. Improved understanding of current health care delivery patterns for these conditions might reveal opportunities to optimize the systems of health care delivery to alleviate child mortality and morbidity. While prior studies have reported variation in patient outcomes and resource use for both complex and routine health conditions according to teaching hospital status, [2-4] and other hospital-level structural characteristics [5-8], no evaluation of variation in patient illness severity and associated resource use burden has been performed among pediatric hospitalizations. This study was conducted to describe patient and hospital characteristics, and hospital resource use, among hospitalizations for pediatric high-impact conditions; and test the hypothesis, based on the authors’ clinical experience and prior work, [9] that the most severely ill children will be hospitalized more often at children’s hospitals and will accrue greater hospital resource use burden than less severely ill children.

## Methods

### Study design

We conducted a retrospective study of children 0–18 years of age hospitalized with one or more of the ten leading causes of child death in 2006. Our data source was the 2006 version of the Kids’ Inpatient Database (KID), developed by the Agency for Healthcare Research and Quality (AHRQ) [10]. The database contains discharge records for nearly 3 million pediatric discharge records obtained from 3,739 hospitals in 38 states. The KID is the only national, all-payer database of hospitalizations for children and contains 80% of the normal non-newborn discharges and 10% of uncomplicated in-hospital births, from participating states. Each record in the KID contains multiple variables, including patient demographics, diagnosis-related groups, up to 15 *International Classification of Diseases, ninth revision, clinical modification* (ICD-9-CM) procedure and diagnosis codes, and vital status at hospital discharge. Each record also includes a sample weight to allow for generation of nationally representative estimates.

### Study sample and variable identification

Hospitalizations for the ten leading causes of child death in 2006 were identified using ICD-9-CM diagnosis codes in either the primary or secondary diagnosis fields, with the subsequent exclusion of diagnoses deemed not amenable to hospital care, including sudden infant death syndrome, homicide and suicide, because these three conditions often result in death prior to arrival in the hospital setting. The ten leading conditions of child death (and their corresponding ICD-9-CM diagnosis codes) in 2006 included: perinatal conditions (760.0-779.9), congenital malformations (740.0-759.9), chronic lower respiratory disease (277.0, 490–496), influenza & pneumonia (480–487), injury (800.00-959.99), malignant neoplasm (140.0-209.9), heart disease (390.0-398.9, 402, 404.9-429.9), benign neoplasm (210.0-239.9), septicemia (038, 995.92, 995.91, 771.81, 995.94, 790.7), and cerebrovascular disease (430–438) [1].

### Study variables

#### Dependent variables

These were measures of hospital resource use, including hospital length of stay (LOS), and total hospital charges. For the analysis, meaningful variables were derived from these original measures of resource use including:

i. Average (mean) hospital LOS.

ii. Average (mean) total hospital charges.

iii. Cumulative annual hospital patient-days.

iv. Cumulative annual hospital charges.

The cumulative annual patient-days were calculated as the product of total number of patients and their LOS, while cumulative annual hospital charges were calculated as the product of total number of patients and their accrued hospital charges. These cumulative annual patient-days and hospital charges were calculated to estimate the overall resource use burden borne by the various types of hospitals in 2006.

#### Independent variables

These were patient and hospital characteristics. Patient characteristics that were investigated included patient *age, gender, insurance payer, and severity of illness.* Patient illness severity was assessed using the All-patient refined diagnosis related group (APRDRG) classification within the KID. The categories of APRDRG-severity of illness were minor, moderate, major, and extreme loss of function, hereafter referred to as *patient illness severity*. The APRDRG severity classification is a proprietary, validated, and extensively used measure of illness severity that utilizes patient discharge data including principal and secondary diagnoses, procedures, and demographic information to assign patients to subclasses of illness severity [11]. Due to small sample size for hospitalizations with minor illness severity, the categories for minor and moderate illness severity were combined for the analyses and referred to as minor-moderate illness severity.

Hospital characteristics of interest in the study included *total bed size* (large, medium, and small) defined by hospital location and teaching status, *the type of hospital* (children’s hospital status and teaching status), *source of admission* (emergency department, clinic, other hospitals, or other healthcare facilities such as long-term care facilities and nursing facilities), and *hospital location* (urban or rural), as reported in publications available from AHRQ [12]. There are three broad categories of hospitals relating to children: Children’s hospital (teaching and non-teaching), non-children’s teaching hospitals, and non-children’s, non-teaching hospitals. Children’s hospitals were identified by the American Hospital Association annual survey of hospitals, and information from the National Association of Children’s Hospitals and Related Institutions. Teaching hospital status was determined by whether a hospital had an American Medical Association-approved residency program, was a member of the Council of Teaching Hospitals, or had a ratio of full-time equivalent interns and residents to beds of .25 or higher [12]. Due to small sample size for admissions to the non-teaching children’s hospitals, children’s teaching and non-teaching hospitals were combined for the analyses. The institutional review board of the University of Michigan medical school approved the study.

### Statistical analysis

After initial identification of the number of hospitalizations with high-impact conditions in the KID, the frequency distribution of these hospitalizations was described according to the patient and hospital characteristics, and separately, according to patient illness severity. Thereafter, the mean hospital LOS and charges, and cumulative annual hospital LOS and charges (with associated 95% confidence intervals) for the hospitalizations were calculated and compared according to the categories of patient illness severity. Bivariate comparisons were performed with the aid of the Chi Square test for categorical data and the Student t-test for continuous data. An alpha of 0.05 was set as the threshold for statistical significance. The number of hospitalizations is presented as unweighted data, while all point estimates and accompanying 95% confidence intervals were calculated using sample weights to account for the complex survey design. All analyses were performed using Stata 10 for Windows (Stata Corp.; College Station, Texas) which accounted for the complex survey design.

## Results

### Sample characteristics

There were an estimated 3,084,548 hospitalizations of children with high-impact conditions in the United States in 2006, comprising 42% of all child hospitalizations during that year. Perinatal conditions were the most common, followed in order by congenital anomalies and chronic lower respiratory disease (Table 1). Most hospitalizations were of infants, with 72% under the age of 1 year, and there was more frequent hospitalization of male than female patients (Table 2). There was variation in illness severity with 84.4% of hospitalizations exhibiting minor-moderate illness severity, 12.2% had major illness severity, and 3.4% had extreme illness severity. Although occurring predominantly at hospitals located in urban areas, hospitalizations were evenly distributed among hospital types based on teaching and children’s hospital status (Table 2). Children were most often admitted from outpatient care settings such as clinics and referring physician offices, compared with other sources of admission. Commercial health plans and the Medicaid program were the predominant insurers for the hospitalizations (Table 2).

**Table 1:** Distribution of hospitalizations for pediatric high-impact conditions in 2006 *

	**%**	**[95% CI]**	
Perinatal conditions	63.1	61.8	64.5
Congenital anomalies	17.7	17.1	18.3
Chronic lower respiratory disease	12.0	11.5	12.5
Influenza & Pneumonia	8.2	7.9	8.5
Injury	7.4	7.1	7.8
Malignant neoplasms	3.2	2.8	3.5
Heart disease	3.0	2.7	3.2
Benign neoplasms	1.3	1.2	1.3
Septicemia	0.9	0.9	1.0
Cerebrovascular disease	0.1	0.1	0.1

* Diagnoses are not mutually exclusive.

CI = Confidence Interval.

**Table 2:** Patient and hospital characteristics of hospitalizations for pediatric high-impact conditions

**Characteristic**	**Number of hospitalizations (%)**	
**Age**		
< 1 year	1,020,642	(71.9)
1-4 years	185,053	(9.9)
5-9 years	112,703	(6.1)
10-14 years	100,192	(5.4)
15-19 years	129,065	(6.7)
**Gender**		
Male	861,677	(55.2)
Female	678,603	(44.8)
**Source of Admission**		
Routine/Office/Clinic	1,056,752	(75.6)
Emergency Department	346,692	(19.2)
Another Hospital	82,818	(4.6)
Another health care facility, including long term care & nursing care facility	9,815	(0.6)
Court/Law enforcement Institution	530	(< 0.1)
**Hospital Bed Size**		
Small	179,272	(12.2)
Medium	399,609	(26.9)
Large	941,176	(60.9)
**Hospital Location**		
Rural	128,951	(9.8)
Urban	1,391,106	(90.2)
**Type of Hospital**		
Children’s Teaching and Non-Teaching Hospital	480,139	(30.5)
Non-Children’s Teaching Hospital	438,665	(30.9)
Non-Children’s Non-Teaching Hospital	533,000	(38.7)
**APRDRG-Levels of Illness Severity**		
Minor-moderate	1,241,632	(84.4)
Major	239,702	(12.2)
Extreme	66,321	(3.4)
**Payer Type**		
Medicaid	692,743	(43.7)
Private/HMO	726,487	(48.3)
Self-Pay	69,952	(4.6)
Other	56,110	(3.4)

APRDRG = All-patient refined diagnostic related groups, HMO = Health maintenance organization.

Evaluation of patient and hospital characteristics according to patient illness severity revealed significant variation across strata of illness severity. For children older than 9 years, there was progressively higher frequency of hospitalizations with increased illness severity (Table 3). There was a significantly higher frequency of inter-hospital transfer among children hospitalized with high (major and extreme) illness severity, compared with hospitalizations of children with minor-moderate illness severity (Table 3). In comparison with rural hospitals, urban hospitals were significantly more likely to hospitalize children with high-impact conditions, a pattern most obvious among the more severely ill children (Table 3).

**Table 3:** Patient and hospital characteristics according to patient illness severity

**Characteristic**	**Number of hospitalizations by severity (%)**						**p**
	**Minor-moderate**		**Major**		**Extreme**		
**Age**							
< 1 year	808,137	(72.6)	167,764	(68.2)	44,741	(66.3)	
1-4 years	156,808	(10.0)	21,744	(9.7)	6,501	(10.3)	
5-9 years	95,181	(6.1)	13,918	(6.3)	3,604	(5.7)	
10-14 years	80,767	(5.1)	15,085	(6.7)	4,340	(6.9)	
15-18 years	100,739	(6.2)	21,191	(9.1)	7,135	(10.8)	0.0001
**Gender**							
Male	689,310	(54.9)	134,378	(56.0)	37,989	(57.3)	
Female	545,134	(45.1)	105,168	(44.0)	28,301	(42.7)	0.0001
**Source of Admission**							
Routine/Office/Clinic	855,444	(77.3)	166,788	(70.4)	34,520	(51.7)	
Emergency Department	292,213	(19.2)	41,078	(18.6)	13,401	(21.4)	
Another Hospital	45,926	(3.0)	21,622	(9.9)	15,270	(24.9)	
Another health care facility, including long term care & nursing care facility	6,433	(0.4)	2,163	(1.0)	1,219	(2.0)	
Court/Law enforcement institution	479	(0.1)	49	(0.1)	2	-	0.0001
**Hospital Bed Size**							
Small	146,625	(12.2)	25,705	(11.7)	6,942	(11.9)	
Medium	324,107	(27.1)	60,101	(26.4)	15,401	(25.0)	
Large	750,443	(60.7)	148,380	(61.9)	42,353	(63.1)	0.5804
**Hospital Location**							
Rural	116,732	(10.7)	11,295	(5.4)	924	(1.6)	
Urban	1,104,443	(89.3)	222,891	(94.6)	63,772	(98.4)	0.0001
**Type of Hospital**							
Children’s Teaching and Non-Teaching Hospital	347,849	(27.0)	94,388	(44.6)	37,902	(63.7)	
Non-Children’s Teaching Hospital	357,053	(31.5)	66,159	(28.8)	15,453	(23.6)	
Non-Children’s Non-Teaching Hospital	460,607	(41.5)	63,578	(26.6)	8,815	(12.7)	0.0001
**Payer Type**							
Medicaid	548,173	(43.0)	110,907	(46.6)	33,663	(51.0)	
Private/HMO	589,224	(49.0)	109,761	(45.6)	27,482	(41.3)	
Self-Pay	59,312	(4.9)	8,893	(3.7)	1,747	(2.6)	
Other	42,907	(3.1)	9,832	(4.2)	3,371	(5.1)	0.0001

HMO = Health maintenance organization.

**Figure 1:**
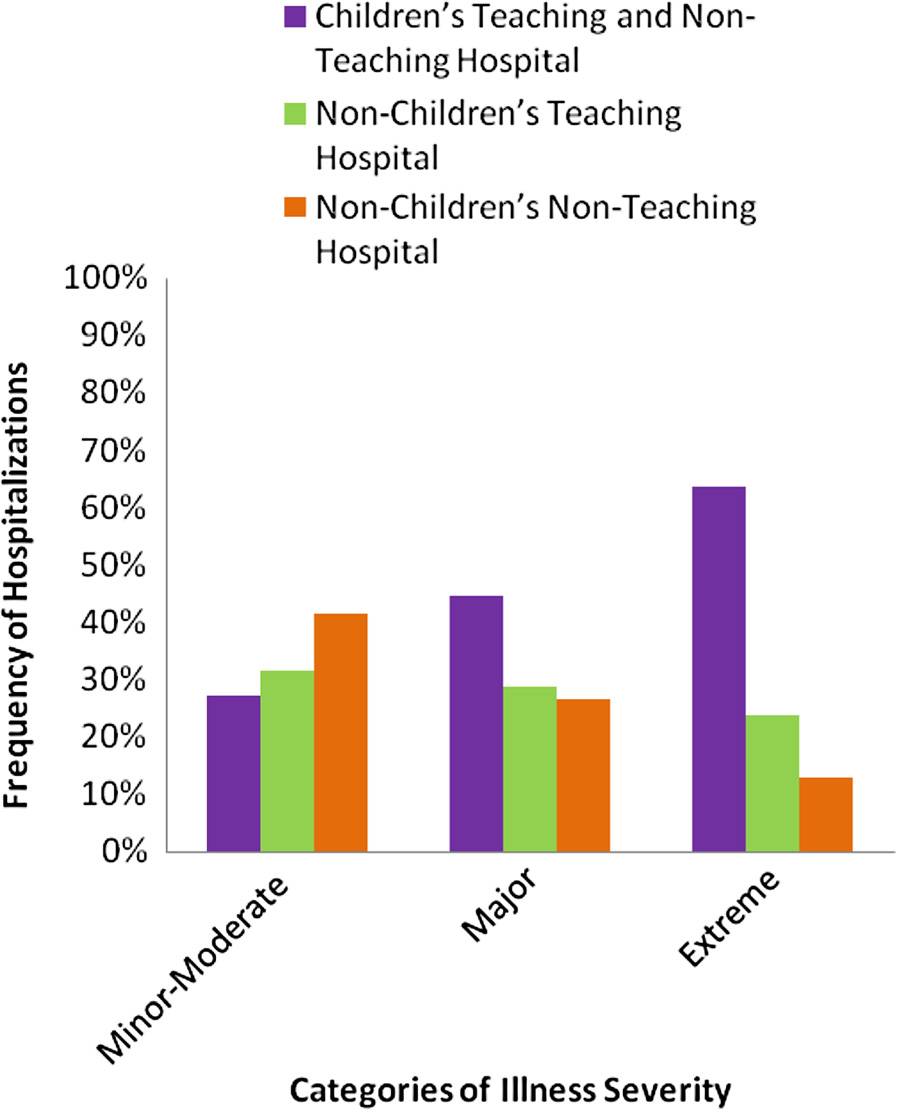
Distribution of Hospitalization By Type of Hospital and Illness Severity.

Importantly, hospitalizations to different hospital types varied significantly with patient illness severity (Table 3). Most of the extremely ill were hospitalized at children’s hospitals, compared with non-children’s teaching hospitals, and hospitals without teaching or children’s hospital status (64% versus 23%, and 13%, respectively, p < .001 ) [Figure 1]. Likewise, children with major illness severity were more likely to be hospitalized at children’s hospitals, versus non-children’s teaching hospitals, and hospitals without teaching or children’s hospital status (45%, versus 29%, and 26%, respectively, p < .001) [Table 3]. Contrariwise, hospitalizations with the lowest illness severity (minor-moderate illness severity) were more likely to be at hospitals without children’s or teaching status, compared with non-children’s teaching hospitals, or children’s hospitals (41%, versus 32%, and 27%, respectively, p < .001). Of note, the Medicaid program appeared to bear increasing burden of insurance coverage with increment in the level of patient illness severity (Table 3).

### Average and Cumulative In-Hospital Resource Use

Comparisons of hospital LOS and mean hospital charges revealed progressive increase in both measures with increase in illness severity. Mean hospital stay was longest among the extremely ill (32.8 days), compared with children with major (9.8 days), or minor-moderate (3.4 days) illness severity (Table 4). Similarly, mean total hospital charges was highest among the extremely ill children ($ 185,518), compared with children exhibiting major ($ 45,371), or minor-moderate ($ 10,483) illness severity (Table 4). Further comparison of mean hospital charges across hospital type, revealed consistently higher hospital charges accruing to hospitalizations within children’s hospitals compared with other types of hospitals (Table 5).

**Table 4:** Average length of stay and hospital charges by patient illness severity

**Characteristic**	**Categories of illness severity**			**p**
	**Minor-moderate**	**Major**	**Extreme**	
**Mean Hospital LOS, days (95% CI)**	3.4	9.8	32.8	< 0.001
	(3.3 – 3.4)	(9.6 – 10.0)	(31.8 – 33.7)	
**Mean Hospital Charges, 2006 US $ (95% CI)**	10,483	45,371	185,518	< 0.001
	(10,018 – 10,948)	(43,252 – 47,490)	(178,577 – 192,458)	

LOS = Length of stay, CI = Confidence Interval.

**Table 5:** Mean hospital resource use by hospital type and patient illness severity in 2006

**Characteristic**	**Hospital type**	**Categories of illness severity**		
		**Minor- moderate**	**Major**	**Extreme**
**Mean Hospital LOS, days (95% CI)**	Children’s Teaching and Non-Teaching Hospital	3.8	10.7	33.1
		(3.7-3.9)	(10.4-11.1)	(31.8-34.4)
	Non-Children’s Teaching Hospital	3.6	10.4	35.5
		(3.5-3.7)	(9.9-10.8)	(33.8-37.2)
	Non-Children’s Non-Teaching Hospital	2.9	7.4	25.5
		(2.8-3.0)	(7.1-7.8)	(23.3-27.7)
**Mean Hospital Charges, 2006 US $ (95% CI)**	Children’s Teaching and Non-Teaching Hospital	18,095	60,124	198,171
		(16,756-19,434)	(56,064-64,184)	(188,071-208,271)
	Non-Children’s Teaching Hospital	9,486	40,584	178,776
		(8,852-10,118)	(37,478-43,690)	(165,652-191,899)
	Non-Children’s Non-Teaching Hospital	6,597	27,389	145,325
		(6,294-6,901)	(25,127-29,651)	(131,371-159,279)

Differential hospitalization of extremely ill children at children’s hospitals resulted in concomitant disparity in resource use burden in 2006, as evidenced by higher cumulative hospital charges among children’s hospitals ($ 7.4 billion) among the extremely ill, compared with non-children teaching hospitals ($ 3.2 billion, p = 0.023), and non-children’s, non-teaching hospitals ($ 1.5 billion, p < 0.001). Similar patterns were observed among children with lower levels of illness severity [Table 6]. Cumulative annual LOS, in patient-days, among the extremely ill was similarly higher in children’s hospitals (1.3 million), compared with non-children’s teaching hospitals (0.7 million, p = 0.030), and hospitals without teaching or children’s status (0.3 million, p < 0.001). Similar resource use patterns were observed among children with major illness severity (Table 6), however, among children with minor-moderate illness severity, hospitals without children’s or teaching status had the highest number of patient-days followed by non-children’s teaching hospitals, and children’s hospitals, in order of decreasing cumulative annual patient stay (Table 6).

**Table 6:** Cumulative hospital resource use by hospital type and patient illness severity in 2006

**Characteristic**	**Hospital type**	**Categories of illness severity**		
		**Minor-moderate**	**Major**	**Extreme**
**Cumulative Hospital Stay, Million Patient-Days (95% CI)**	Children’s Teaching and Non-Teaching Hospital	2.2	1.2	1.3
		(1.8-2.5)	(1.1-1.4)	(1.1-1.4)
	Non-Children’s Teaching Hospital	2.5	0.9	0.7
		(2.3-2.7)	(0.8-1.0)	(0.6-0.8)
	Non-Children’s Non-Teaching Hospital	2.8	0.6	0.3
		(2.6-2.9)	(0.6-0.7)	(0.2-0.3)
**Cumulative Hospital Charges, 2006 US Billion $ (95% CI)**	Children’s Teaching and Non-Teaching Hospital	10.3	7.1	7.4
		(8.7-11.8)	(5.9-8.2)	(6.3-8.6)
	Non-Children’s Teaching Hospital	6.3	3.4	3.2
		(5.6-7.0)	(2.9-3.8)	(2.7-3.7)
	Non-Children’s Non-Teaching Hospital	6.2	2.3	1.5
		(5.8-6.6)	(2.0-2.5)	(1.3-1.8)

CI = Confidence Interval.

## Discussion

The study highlights important findings about hospitalizations for pediatric conditions of great public health significance given their notoriety as the leading causes of child death among U.S. children. These high-impact hospitalizations are frequent, comprising 42% of all child hospitalizations in the U.S. in 2006. Majority of hospitalizations were of minor-moderate illness severity, were predominantly within urban hospitals, without significant variation in the overall frequency of hospitalizations by the teaching or children’s hospital status of the treating hospitals. Of importance, however, stepwise increment in levels of patient illness severity was associated with greater likelihood of hospitalization at children’s hospitals, compared with other hospital types. Elevated patient illness severity was also associated with inter-hospital patient transfer, greater likelihood of hospitalization within hospitals located in urban areas, and insurance coverage by the Medicaid program. Differential hospitalization of patients according to their illness severity was highly correlated with concomitant variation in hospital resource use burden.

In aggregate, the frequency of hospitalizations did not vary by the type of hospital, however; a pattern of differential hospitalization was observed according to patient illness severity, with most of the children with heightened illness severity receiving care at children’s hospitals. As previously reported, [12] this observation suggests a practice of pre-hospital patient triage according to illness severity, with funneling of the most severely ill patients to children’s hospitals where specialized care might be more readily available. Elevated patient illness severity, which might be a marker of disease progression despite ongoing health care, was also associated with increased likelihood of inter-hospital transfer, most often to a children’s hospital. Hospitalizations involving inter-hospital transfer deserve further investigation given this finding which corroborates prior reports which have described an association between interhospital transfer and both heightened illness severity and elevated hospital resource use [12-14].

In an era of scarce health care resources and a push for accountable health care, it is important to better understand resource use for these high-impact conditions and how it varies by the type of treating hospital. Such characterization might provide insights into hospital-level variation in health care resource use for pediatric hospitalizations as a whole. This study revealed significant variation in resource use according to both the type of hospital where children received care, and the severity of patient illness. Unsurprisingly, extremely ill children had much longer average hospital stay and higher average hospital charges, when compared with children with lower illness severity. Further, among hospitalizations with either major or extreme illness severity, children’s hospitals and non-children’s teaching hospitals had significantly longer cumulative annual hospital stay (in patient-days) when compared to hospitals without teaching or children’s hospital status, a reflection of higher census of hospitalizations with elevated illness severity at the more specialized hospitals.

Among hospitalizations with lower illness severity, however; there was an opposite observation, as hospitals without teaching or children’s status had the highest number of patient-days compared with other types of hospitals. This is an important finding as it reflects a divergence of hospital resource use by type of hospital, with the more severely ill utilizing more patient-days at more specialized hospitals, while the less specialized hospitals have a higher resource use accruing to less severely ill patients. This finding is novel because, while a previous study among hospitalizations for common pediatric conditions highlighted variation in the duration of hospitalization by hospital type, the authors were unable to attribute the observed variation to any specific factors [7]. A significant limitation of that study, however, was the inability to describe and account for patient illness severity, an important confounder of the relationship between hospital type and resource use. As reported in the current study, disparate hospital resource use appeared to be highly correlated with patient illness severity. Despite this important finding, however; it is important to note that outside of illness severity, multiple patient characteristics, practice patterns, or institutional factors, may cause wide differences across hospitals in the duration of hospital stay, [15] and should be further explored in a systematic fashion to develop efficient health care delivery systems that also enhance patient outcomes.

Cumulative annual hospital charges also varied by hospital type and patient illness severity. Hospitalization of children with high (major or extreme) illness severity within children’s hospitals and non-children’s teaching hospitals accrued higher charges than at hospitals without teaching or children’s hospital status. Although the frequency of hospitalizations for children with low (minor-moderate) illness severity was higher among hospitals without teaching or children’s hospital designation than other hospital types; the pattern of differential charges persisted among this lower illness severity group, with accrual of higher charges at the more specialized hospitals.

It is unknown why hospital charges that accrued to high-impact hospitalizations were higher the more specialized the hospital, regardless of patient illness severity. Various theories have been expounded to explain why children’s and teaching hospitals might accrue more charges than hospitals without teaching or children’s designations. It has been previously speculated that sub-specialty care and use of advanced technology within these latter specialized hospitals might explain some of the differential charges reported [6]. Higher charges have also been reported to accrue among hospitalizations in teaching versus non-teaching hospitals presumably as a result of specialization of medical care, innovations in care, and medical education. [16,17] Future study is warranted to investigate in-hospital resource use and the associated patient- and hospital-level outcomes for children hospitalized with high-impact conditions.

Although commercial insurance plans and the Medicaid program were the principal insurance payers for hospitalizations with high-impact pediatric conditions overall; the Medicaid program bore greater burden of coverage when compared with other insurance payers, with increasing patient severity. Insuring more than half of the extremely ill children and shouldering the burden of increasing numbers of patient as severity of illness worsened; this observation of elevated illness severity among the Medicaid-insured children might be a reflection of the program’s mission to provide coverage for children more likely to be indigent, with comorbid illness, and presumably poor access to the health care system, with resultant high illness severity upon hospitalization [18,19]. The finding highlights the safety net nature of the Medicaid program that raises the concern for worse outcomes for children if the existence of this and similar programs was jeopardized [20,21]. Further research is warranted to elucidate any modifiable determinants of child health and illness that might contribute to the described characteristics of pediatric high-impact hospitalizations, and variation in hospital resource use.

The study finding of disparate hospitalization of the most severely ill children to children’s hospitals might have implications for health care policy makers. It suggests the critical importance of in-depth assessment of patient illness severity among hospitalized children, and incorporation of such assessment into measures of child health outcomes and hospital resource use. Further, patient severity measures should be included in appropriate risk-adjustment of benchmark measures used for inter-institutional comparison of hospital performance and resource use to ensure that hospitals which care for very severely ill children ab initio, or on inter-hospital transfer, are not penalized for doing so, by policy-makers and insurance payers.

The findings of the current study should be interpreted in light of certain limitations. The KID is a database of administrative discharge data without clinical information beyond what can be captured in ICD-9-CM diagnosis and procedure codes. Hence, it was not possible to study the clinical course for each patient, and how clinical care was coordinated at the patient and hospital levels, within the hospitals. Also, the KID has no follow-up or longitudinal information on patients after hospital discharge. Therefore, determination could not be made of the long term fatal or non-fatal outcomes for survivors of these leading conditions of child mortality in the U.S.

## Conclusion

Gradation of increasing illness severity among hospitalized children with high-impact conditions was associated with concomitant prolongation of the duration of hospitalization and higher hospital charges. The study findings have significant implications for children’s and urban hospitals which appear to accrue the highest resource use burden due to the preferential hospitalization of the most severely ill children ab ibnitio, or on inter-hospital transfer, to these hospitals. This important role of children’s hospitals warrants recognition by hospital administrators and policy-makers. The Medicaid program appeared to serve the role of a safety net program insuring more than half of the extremely ill children and shouldering the burden of increasing numbers of patient as severity of illness worsened. Efforts to ensure continued existence of the program and similar safety net programs, and enhance access to them, will likely have a salutary effect on child health outcomes for conditions of high public health significance.

## Abbreviations

KID: Kid’s inpatient database; ICD-9-CM: International classification of diseases, ninth revision, clinical modification; APR-DRG: All patient refined diagnostic-related groups.

## Competing interests

The authors have no competing interests to disclose.

## Authors’ contributions

FO made substantial contributions to the intellectual content of the paper by participating in the conception and design, data acquisition, interpretation of the data, drafting of the manuscript, critical revision of the manuscript for important intellectual content, and providing supervision. RM made substantial contributions to the intellectual content of the paper by participating in the interpretation of the data, drafting of the manuscript, and critical revision of the manuscript for important intellectual content. AG made substantial contributions to the intellectual content of the paper by participating in the analysis and interpretation of the data, providing statistical expertise, and in critical revision of the manuscript for important intellectual content. All authors read and approved the final manuscript.

## Funding

No funding was obtained for the study.

## Pre-publication history

The pre-publication history for this paper can be accessed here:

http://www.biomedcentral.com/1471-2431/12/61/prepub
